# A real-world study of adverse drug reactions of two isocitrate dehydrogenase inhibitor based on the US FDA adverse event reporting system and VigiAccess databases

**DOI:** 10.3389/fphar.2024.1489045

**Published:** 2024-11-07

**Authors:** Mengmeng Peng, Qian Guo, Zihan Dang, Baiquan Zhang, Manjuan Li, Zixuan Wang, Xuemian Lu, Jie Lin

**Affiliations:** ^1^ Department of Endocrinology, The Third Affiliated Hospital of Wenzhou Medical University, Wenzhou, China; ^2^ Wenzhou Key Laboratory for the Diagnosis and Prevention of Diabetic Complication, Wenzhou, China; ^3^ Department of Rhinolohy, The First Affiliated Hospital of Zhengzhou University, Zhengzhou, China; ^4^ Department of Health Studies and Applied Educational Psychology, Columbia University, New York, NY, United States; ^5^ Department of Respiratory Medicine, The First Affiliated Hospital of Zhengzhou University, Zhengzhou, China; ^6^ Department of Pharmacy, The Third Affiliated Hospital of Wenzhou Medical University, Wenzhou, China

**Keywords:** pharmacovigilance, isocitrate dehydrogenase inhibitor, adverse drug reaction, WHO-VigiAccess, AML

## Abstract

**Background and objectives:**

Isocitrate dehydrogenase (IDH) inhibitor drugs (Enasidenib, Ivosidenib) restore normal metabolism and epigenetic regulation in cells, offering a precision-targeted therapeutic option for acute myeloid leukemia (AML) patients with IDH mutations by specifically inhibiting mutated IDH enzymes. This research evaluates the relationship between adverse drug reactions (ADR) and the use of two isocitrate dehydrogenase inhibitors by using the database from the World Health Organization (WHO) VigiAccess and compares the characteristics of ADRs of the two drugs.

**Methods:**

This study design used the retrospective descriptive analysis. We calculated the ratio of ADRs recorded in reports to compare the same points and different points in ADRs between two medications. Proportional reporting ratio (PRR) and reported odds ratio were used to evaluate the relationship between these two isocitrate dehydrogenase inhibitor medications and adverse events.

**Results:**

Overall, during the search, 4,072 adverse events related to two types of isocitrate dehydrogenase inhibitors were reported in VigiAccess. The results revealed that the top 10 most common AEs were off label use, death, fatigue, nausea, diarrhea, acute myeloid leukemia, drug ineffective, differentiation syndrome, platelet count decreased and decreased appetite. Compared two drugs, enasidinib had the highest adverse reaction reporting rate in general disorders and administration site conditions while ivosidenib had the highest adverse drug reactions reporting rate in injury, poisoning and procedural complications.

**Conclusion:**

Based on the current comparative observational studies, the ADR reports received by the World Health Organization, Food and Drug Administration for these drugs list common and specific adverse drug reactions. Clinical doctors should develop individualized treatment plans based on the adverse reactions of different drugs and the specific conditions of patients to promote the rational use of these expensive medications.

## 1 Introduction

Acute myeloid leukemia is a malignant disease of myeloid hematopoietic stem cells. The disease is characterized by abnormal proliferation of primitive and immature myeloid cells in bone marrow and peripheral blood (Wang et al., 2021). AML is the most common type of acute leukemia in adults. With a relatively short survival rate of only 23.6% at 5 years, AML threatens the people’s health seriously (Villamón et al., 2018). The median age of AML diagnosis is 68–71 years old showing that AML mostly occurs in the elderly and the incidence of AML increases with age. Therefore, with the aggravation of population aging, it is expected that the incidence of AML will continue to increase gradually. At present, there are two main classification methods for acute myeloid leukemia: FAB classification and World Health Organization (WHO) classification. The WHO classification, also known as MICM classification, is based on morphology, immunology, cytogenetics and molecular biology which is relatively complex (Chengxin Luan et al., 2015). FAB classification is an easier and classic classification method for AML. AML is mainly divided into eight types from M0 to M7: acute myeloblastic leukemia without maturation (M0), acute myeloblastic leukemia with minimal maturation (M1), acute myeloblastic leukemia with maturation (M2), acute promyelocytic leukemia (M3), acute myelomonocytic leukemia (M4), acute monocytic leukemia (M5), acute erythroleukemia (M6), and acute megakaryoblastic leukemia (M7) (Kajsa Paulsson et al., 2001).

The pathogenesis of AML remains unclear, but various factors have been found to be related to its onset. Previous studies have found that the pathogenesis of AML is related to changes in genes which participate in cell metabolism and epigenetic regulation. Mutations in isocitrate dehydrogenase (IDH)1 and IDH2 are found in 6%–16% and 8%–19% of patients with acute myeloid leukemia (AML), respectively (Bruno et al., 2016). The enzymes encoded by IDH1 and IDH2 genes are widely present in the human body. The IDH1 protein plays a role in the cytoplasm and peroxisomes. The IDH2 protein is an important enzyme of the tricarboxylic acid (TCA, also called the “citric acid” or Krebs) cycle. IDH2 and IDH1 proteins catalyze the oxidation and decarboxylation of isocitrate to α - ketoglutarate (α-KG) to produce reduced nicotinamide adenine dinucleotide phosphate (NADPH) from NADP+ (Martelli et al., 2020). But the mutant IDH1/2 (mIDH1/2) enzyme catalyzes the reduction of α-KG to tumor metabolite d-2-hydroxyglutarate (2-HG). 2-HG accumulation leads to DNA hypermethylation through competitive inhibition of α - ketoglutarate dependent dioxygenases. These epigenetic changes are hypothesized to be the main driving factors for myeloid differentiation arrest, which is a mark of AML.

Supported by strong genetic theoretical foundations and biological evidence that IDH mutations play a critical role in driving leukemia development, extensive researches have been conducted on the development of IDH mutant targeted drugs. IDH inhibitors can specifically bind to mutated IDH enzymes to inhibit their activity, thereby preventing the conversion of α - KG to 2-HG by IDH enzymes and reducing the accumulation of 2-HG in cells. As the level of 2-HG decreases, the activity of inhibited α - KG dependent dioxygenases is restored. These enzymes can remove methylation modifications on DNA and histones to restore normal epigenetic regulation. The first compounds received clinical concept validations for the treatment of IDH1 and IDH2 mutant AML are enasidenib and ivosidenib.

Although isocitrate dehydrogenase inhibitor drugs have shown significant efficacy in treating AML, long-term use of isocitrate dehydrogenase inhibitor drugs may lead to serious adverse reactions. A meta-analysis showed that common adverse reactions included nausea, vomiting, blood bilirubin increased, diarrhea, constipation, anemia, decreased appetite, electrocardiogram QT prolongation, fatigue, dyspnea, rash, dysgeusia and leukocytosis. Severe cases may result in neutropenia, thrombocytopenia, sepsis, pneumonia and differentiation syndrome. A study conducted in 2020 showed that differentiation syndrome (DS) was most relevant, potentially life-threatening side effect for patients with ivosidenib and enasidenib (Del Principe et al., 2019).

The occurrence of ADRS not only brings economic burden to patients, but also affects their quality of life and serious adverse reactions can even endanger their lives. Therefore, clinical doctors must thoroughly understand these potential adverse reactions and reduce their impacts through close monitoring and timely management.

Although clinical test is an indispensable part to determine the efficacy of new drugs and identify common ADRs, they may not be able to access all situations in real world because rare and serious events may only occur in clinical settings after widespread use of drugs. However, the pharmacovigilance (PV) analysis which involves monitoring and evaluating the safety of the drugs has solved this problem.

Despite the intrinsic limitations, spontaneous reporting systems (SRS) represent a valuable source to obtain real-world data about the safety profile of drugs and vaccines, compare therapeutic options, and gain insight into the potential mechanisms of ADRs (Hazell and Shakir, 2006). SRS is mainly used to detect the discovered ADRs at early stage. It can continuously monitor the safety of drugs through the data collected by SRS and take necessary measures to reduce risks in time which is of great significance for protecting public health and guiding clinical practice.

This study retrieved two isocitrate dehydrogenase inhibitor drugs approved by the US Food and Drug Administration (FDA): Enasidenib and Ivosidenib. Although these two types of isocitrate dehydrogenase inhibitors have been approved for clinical use, there have been few studies comparing the common points and different points in the ADRs caused by these two drugs currently. This study not only comprehensively evaluates the safety of drugs, but also has significant implications for guiding clinical medication.

## 2 Materials and methods

### 2.1 Drug samples

Table 1 presents the basic information of the two isocitrate dehydrogenase inhibitor drugs that are available for clinical treatment in our study.

**TABLE 1:** The basic Information of the two isocitrate dehydrogenase inhibitor drugs studied for clinical treatment.

Drug name and brand name	Structure	Main conditions	First marketing time	Biosimilars
Enasidenib Idhifa^®^	IDH2 inhibitor	Recurrent or refractory acute myeloid leukemia	2017	LuciEna, Enacitib
Ivosidenib Tibsovo^®^	IDH1 inhibitor	Recurrent or refractory acute myeloid leukemia, newly diagnosed acute myeloid leukemia and cholangiocarcinoma	2018	

Enasidenib is the world’s first approved IDH2 inhibitor which generates clinical reactions in 40% of relapsed or refractory AML (R/R AML) patients by promoting leukemia cell differentiation. In August 2017, enasidenib was approved by the US FDA and used to treat relapsed or refractory acute myeloid leukemia with IDH2 mutations (Schenkein, 2018). The milestone experiment of enasidenib was published by Stein et al. (2017) which established the pharmacokinetic and pharmacodynamic characteristics of enasidenib for the first time and showed its clinical efficacy in R/R AML patients.

Ivosidenib is the world’s first approved potent oral targeted inhibitor for IDH1 mutant cancer. The drug was approved by the US FDA in August 2021 for the treatment of patients with AML and locally advanced or metastatic cholangiocarcinoma. Meanwhile, ivosidenib is the first mutated IDH1 enzyme inhibitor to obtain clinical concept validation in human trials. Biochemical and cellular biology analysis showed that ivosidenib inhibited several IDH1-R132 mutants and exhibited high selectivity towards the IDH1 subtype. In cell-based experiments, ivosidenib demonstrated good cellular efficacy in various IDH1-R132 endogenous and overexpressing cell lines. The good pharmacokinetic characteristics and good tolerability of these preclinical data provide a basis for promoting the clinical development of ivosidenib. Ivosidenib mainly works by reducing the carcinogenic metabolite 2-hydroxyglutarate (2-HG) produced after mutation. This inhibitor does not directly kill cells, but induces malignant cell differentiation to treat cancer.

Up to August 2024, there are two biosimilar of enasidib. There is no biosimilar of ivosidenib on the market currently. In recent research, Celgene and Agios Pharmaceuticals have jointly developed vorasidenib, a broad-spectrum IDH1/IDH2 inhibitor that can simultaneously inhibit IDH1 and IDH2 mutations. Vorasidenib is intended for the treatment of malignant solid tumors and malignant hematological tumors. On 9 August 2024, the US FDA approved vorasidenib for postoperative treatment of grade 2 astrocytomas or oligodendrogliomas in adults and children aged 12 years and older post operative treatment (including biopsy, subtotal resection, or total resection) carrying isocitrate dehydrogenase 1 or 2 mutations.

A Phase-1 research evaluated the safety and efficacy of ivosidenib or enasidenib combined with intensified chemotherapy in newly diagnosed mIDH1/2 AML patients. The results showed that the combination therapy had good safety and significant efficacy during induction and consolidation therapy. This indicated that enasidinib and ivosidib could be used in combination in specific situations.

### 2.2 Data source

WHO-VigiAccess retrieved all adverse events reported after the clinical use of isocitrate dehydrogenase inhibitor medications on 10 August 2024. The webpage that users can log in is https://www.vigiaccess.org. Data collected in WHO-VigiAccess covers age, sex, continents and reporting years. WHO-VigiAccess is a free portal for PIDM database, allowing to search drug safety reports received by UMC. This definition relies on the System Organ Classification (SOC) and Preferred Terms (PTs) of the Medicine Regulating Activity (MedDRA) Dictionary (Li et al., 2023). To characterize the toxicity spectrum, we retrieved data for each drug and identified all adverse events based on the recorded MedDRA SOC at pt levels. MedDRA uses reporting terms from several dictionaries, such as the World Health Organization Adverse Reaction Terminology (WHO ART). We chose 20 elements directly related to disease symptoms from the 27 elements in SOC classification for analysis. We divided the data into groups by using outcome codes to study the detected safety signals, resulting in three severity categories: death, hospitalization and major events including life-threatening events, disability and congenital anomaly.

### 2.3 Disproportionality analysis

Disproportionation analysis is a data mining method, which is mainly used to evaluate the correlation between drugs and adverse reactions. The core principle is to use a 2 × 2 contingency table to compare the frequency of adverse events observed in the exposed group and the non-exposed group, so as to quantify the association between drugs and adverse events. When the proportion of AEs in the exposed group exceeded that in the unexposed group, it was inferred that there was an association between drugs and specific AEs, indicating the presence of a disproportionation signal. After exceeding the threshold, the larger the signal value, the stronger the signal. In this study, we used two disproportional analysis methods: reported odds ratio (ROR) and proportional reporting ratio (PRR) to evaluate the possible association between eflornithine, selumetinib and AEs under general disease and administration site conditions. ROR is mainly used to measure the imbalanced probability of reporting AES for specific drugs compared with other drugs.

The calculation formula was:

$$ROR=\frac{a\times d}{b\times c}$$

(a) refers to the quantity of reports for particular drugs and particular AEs, (b) represents the quantity of reports for specific drugs and other AEs, (c) refers to the number of reports on other drugs and specific AEs (d) represents the number of reports on other drugs and other AEs.

PRR refers to the proportion of spontaneous reports of a specific drug associated with a specific adverse outcome divided by the corresponding proportion of other drugs. The calculation formula was:

$$PRR=\frac{a\times \left(c+d\right)}{c\times \left(a+b\right)}$$

Both ROR and PRR require that at least 5 cases (a ≥5) of particular drug and AEs to consider the calculated results valid.

If the ROR = 1: No signal exists; the ADR of interest is as common with the drug of interest as with other drugs. If the ROR <1: No signal exists; the ADR of interest is less frequent with the drug of interest than with other drugs. If the ROR >1: The ADR of interest is more frequent with the drug of interest than with other drugs; there is thus a pharmacovigilance signal, and the higher the ROR, the greater the disproportionality (Montastruc et al., 2011).

In our analysis, we systematically evaluate the ratio of ADRS reports of using isocitrate dehydrogenase inhibitor drugs in general disorders and administration site conditions. The analysis results help to provide guidance for the correct use of drugs.

### 2.4 Statistical analysis

This study design used the retrospective descriptive analysis. Using Excel descriptive analysis, we researched the features of victims of ADRs caused by using two types of isocitrate dehydrogenase inhibitors from the perspectives of current situation, case reports, case series analysis and data analysis. ADR reporting rate was defined as the quantity of ADR symptoms divided by the total quantity of ADR reports. The frequent ADRs of various drugs were defined as the top 20 symptoms with the highest ADR reporting rate. We calculated the incidence of ADR symptoms reported for each drug and conducted descriptive comparative analysis. We classified the descriptive variables by using rate and percentage.

## 3 Results

### 3.1 Case description of the study

According to the WHO VigiAccess data, the earliest ADRs of enasidenib and ivosidenib were received in 2018 and 2017 respectively. As of 2024, the World Health Organization has received a total of 2,776 adverse reports on enasidib and 1,296 adverse reports on ivosidib, totaling 4,072. There are 4,300 AEs for enasidinib and 3,027 AEs for ivosidinib in these ADR reports. Among the 4,072 reports related to the two types of isocitrate dehydrogenase inhibitors shown in Table 2, excluding 1,219 reports of unknown gender, there are 1,266 reports of adverse reactions in females and 1,587 in males with male-to-female ratio of 1:1.25, no significant gender difference. In addition to the report of unknown age, the age group with the highest reporting incidence rate is ≥75 years old, mainly the elderly. Most AE reports come from the United States (87.3%), next is Europe (11.3%). Table 2 also covers the reporting years for enasidenib and ivosidenib. In the past 8 years, enasidenib had a higher incidence of ADR in 2019 and 2021 than in other years; The incidence of ADR for ivosidenib was higher in 2020 and 2021 than in other years. The incidence of adverse reactions to enasidib has decreased since 2021.

**TABLE 2:** Characteristics of adverse reaction reports of two isocitrate dehydrogenase inhibitor drugs.

	Enasidenib	Ivosidenib
Number of ADR reports	2,776	1,296
Female	1,132 (40.8%)	134 (10.3%)
Male	1,480 (53.3%)	107 (8.3%)
Unknown	164 (5.9%)	1,055 (81.4%)
28 days to 23 months	1 (0.0%)	
2–11	3 (0.1%)	
12–17	2 (0.1%)	1 (0.1%)
18–44	39 (1.4%)	18 (1.4%)
45–64	375 (13.5%)	63 (4.9%)
65–74	527 (19.0%)	62 (4.8%)
≥75	660 (23.8%)	54 (4.2%)
Unknown	1,169 (42.1%)	1,098 (84.7%)
Americas	2,418 (87.1%)	1,136 (87.7%)
Asia	1 (0.0%)	2 (0.2%)
Europe	313 (11.3%)	149 (11.5%)
Oceania	44 (1.6%)	9 (0.7%)
2017		1 (0.1%)
2018	350 (12.6%)	2 (0.2%)
2019	558 (20.1%)	176 (13.6%)
2020	454 (16.4%)	320 (24.7%)
2021	479 (17.3%)	496 (38.3%)
2022	338 (12.2%)	76 (5.9%)
2023	323 (11.6%)	135 (10.4%)
2024	274 (9.9%)	90 (6.9%)

### 3.2 Distribution of 20 SOCs for two isocitrate dehydrogenase inhibitor drugs

Table 3 shows the reporting rates of 20 types of SOCs for two types of isocitrate dehydrogenase inhibitor drugs. Enasidib has the highest reporting rate (40.9%) under general disorders and administration site conditions and a higher reporting rate of adverse reactions (17.8%) in investigations. The ADR reporting rate of ivosidenib is the highest in injury poisoning and procedural complications (51.2%) and the reporting rate is higher in general disorders and administration site conditions (38.7%). The top five types of adverse events (AE) caused by isocitrate dehydrogenase inhibitors are: general disorders and administrative site conditions (1,637 cases, 40.2%), injury poisoning and procedural complications (924 cases, 22.7%), investigations (803 cases, 19.7%), gastrointestinal disorders (685 cases, 16.8%), neoplasms benign malignant and unspecified incl cysts and polyps (452 cases, 11.1%).

**TABLE 3:** ADR number and report rate of 20 SOCs of two isocitrate dehydrogenase inhibitor drugs.

System organ class	Enasidenib (N = 2,776)	Ivosidenib (N = 1,296)
Blood and lymphatic system disorders	152 (5.5%)	106 (8.2%)
Cardiac disorders	51 (1.8%)	53 (4.1%)
Congenital familial and genetic disorders	2 (0.1%)	6 (0.5%)
Ear and labyrinth disorders	11 (0.4%)	13 (1.0%)
Endocrine disorders	2 (0.1%)	3 (0.2%)
Eye disorders	21 (0.8%)	23 (1.8%)
Gastrointestinal disorders	394 (14.2%)	291 (22.5%)
General disorders and administration site conditions	1,135 (40.9%)	502 (38.7%)
Hepatobiliary disorders	53 (1.9%)	21 (1.6%)
Immune system disorders	26 (0.9%)	13 (1.0%)
Infections and infestations	303 (10.9%)	129 (10.0%)
Injury poisoning and procedural complications	261 (9.4%)	663 (51.2%)
Investigations	493 (17.8%)	310 (23.9%)
Metabolism and nutrition disorders	147 (5.3%)	81 (6.3%)
Musculoskeletal and connective tissue disorders	125 (4.5%)	120 (9.3%)
Neoplasms benign malignant and unspecified incl cysts and polyps	321 (11.6%)	131 (10.1%)
Nervous system disorders	232 (8.4%)	176 (13.6%)
Pregnancy puerperium and perinatal conditions	2 (0.1%)	1 (0.1%)
Product issues		13 (1.0%)
Psychiatric disorders	70 (2.5%)	56 (4.3%)
Renal and urinary disorders	64 (2.3%)	31 (2.4%)
Reproductive system and breast disorders	2 (0.1%)	5 (0.4%)
Respiratory thoracic and mediastinal disorders	155 (5.6%)	98 (7.6%)
Skin and subcutaneous tissue disorders	129 (4.6%)	104 (3.4%)
Social circumstances	4 (0.1%)	9 (0.3%)
Surgical and medical procedures	91 (3.3%)	45 (1.5%)
Vascular disorders	54 (1.9%)	24 (0.8%)

In the ADR reported by SOC, there are 5 cases of enasidenib and 7 cases of ivosidenib with an incidence rate exceeding 10%.

### 3.3 Disproportionality analysis based on general disorders and administration site conditions

By observing and comparing the SOC distribution of two types of isocitrate dehydrogenase inhibitors, it was found that under general disease and administration site conditions, the two drugs had the highest reported rates of adverse reactions. To further compare these two medications, we conducted disproportionate analysis using ROR and PRR methods. Table 4 showed that through disproportionate analysis, we found that the ROR values of the two drugs were: Enasidenib: 1.65 (1.48–1.83), Ivosidenib: 0.61 (0.55–0.67). The PRR values of the two drugs were: Enasidenib: 1.49 (1.49–1.83), Ivosidenib: 0.67 (0.55–0.67). The results indicated that Enasidib seemed to be more prone to causing general disease and administration site conditions than Ivosidib.

**TABLE 4:** Disproportionality analysis based on general disorders and administration site conditions.

	ROR (95%CI)	PRR (95%CI)
Enasidenib	1.65 (1.48–1.83)	1.49 (1.49–1.83)
Ivosidenib	0.61 (0.55–0.67)	0.67 (0.55–0.67)

### 3.4 The most common ADRs of two isocitrate dehydrogenase inhibitor drugs

The 20 most common adverse reactions of two types of isocitrate dehydrogenase inhibitors are shown in Table 5. The listed performance is the preferred choice within SOC. The common adverse reactions of all two types of isocitrate dehydrogenase inhibitors include drug ineffective platelet count decreased, asthenia, fatigue, vomiting, dyspnoea, diarrhoea, differentiation syndrome, nausea, off label use and constipation. Compared with the two drugs, enasidib has the highest reported adverse reaction rate of death, while ivosidib has the highest reported adverse reaction rate of 42.8% due to off label use. The top 20 adverse reactions in the report are mostly self-limiting, but there are also some adverse reactions that need attention, such as differentiation syndrome, platelet count decreased and death.

**TABLE 5:** Top 20 ADRs of isocitrate dehydrogenase inhibitor drugs.

Enasidenib (N=2,776)		Ivosidenib (N=1,296)	
ADR	Report rate %	ADR	Report rate %
Death	17.3%	Off label use	42.8%
Off label use	5.7%	Fatigue	9.8%
Nausea	5.6%	Diarrhoea	6.6%
Fatigue	5.6%	Nausea	6.1%
Acute myeloid leukaemia	4.9%	Disease progression	5.8%
Diarrhoea	3.9%	Product dose omission issue	5.8%
Decreased appetite	3.5%	Drug ineffective	3.9%
Drug ineffective	3.4%	Differentiation syndrome	3.9%
Platelet count decreased	3.3%	Platelet count decreased	3.5%
Differentiation syndrome	3.3%	Asthenia	3.2%
Hospitalisation	3.1%	Electrocardiogram qt prolonged	2.9%
Asthenia	2.8%	Product use issue	2.9%
Pyrexia	2.4%	Arthralgia	2.9%
Full blood count decreased	2.1%	Headache	2.7%
Pneumonia	2.0%	Constipation	2.6%
Vomiting	1.8%	Vomiting	2.6%
Dyspnoea	1.8%	Haemoglobin decreased	2.5%
White blood cell count decreased	1.7%	Dizziness	2.5%
Constipation	1.5%	Dyspnoea	2.5%
Rash	1.5%	Pain	2.5%

At the same time, it is important to strictly follow the instructions when using ivosidenib and do not overdose to reduce the occurrence of adverse reactions.

### 3.5 Serious AEs of two isocitrate dehydrogenase inhibitors drugs

By using the database, we can also identify the main adverse events of isocitrate dehydrogenase inhibitor drugs, including death, hospitalization, life-threatening events, disability and congenital anomaly.

**FIGURE 1:**
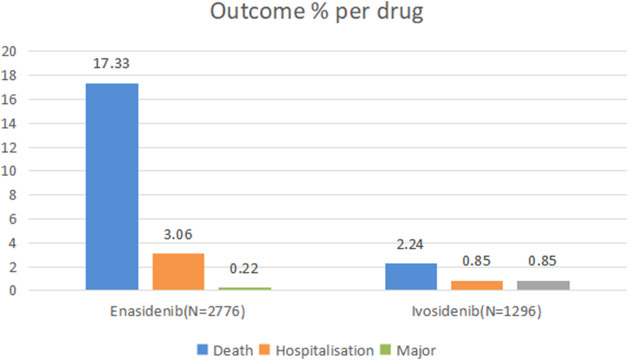
Outcomes for serious adverse events associated with isocitrate dehydrogenase inhibitor drugs at the preferred term level (life-threatening events, disability and congenital anomaly).

The proportion of serious adverse reactions to enasidib and ivosidib was 20.61% and 3.94%, respectively (Figure 1).

### 3.6 The same and different points of common ADRs of two isocitrate dehydrogenase inhibitors drugs

By comparing the top 20 ADRs reported by two isocitrate dehydrogenase inhibitor drugs in SOC, we found a total of 169 common adverse reactions at the PTs level for both drugs. Table 6 lists all the commonalities. The most frequent ADRs of the two drugs are blood and lymphatic system disorders, with the top five reported being cytopenia, febrile neutropenia, neutropenia, anaemia, haematotoxicity, followed by is cardiac Disorders, with the top five reported being cardiac failure congestive, cardiac failure, cardiac disorder, atrial fibrillation, palpitations.

**TABLE 6:** Same ADRs of two isocitrate dehydrogenase inhibitors drugs.

System organ classes	ADRs	Signal N
Blood and lymphatic system disorders	Cytopenia, Febrile neutropenia, Neutropenia, Anaemia, Haematotoxicity, Hyperleukocytosis, Lymphadenopathy, Leukocytosis, Pancytopenia, Thrombocytopenia, Blood disorder	11
Cardiac disorders	Cardiac failure congestive, Cardiac failure, Cardiac disorder, Atrial fibrillation, Palpitations, Myocardial infarction	6
Congenital, familial and genetic disorders		
Ear and labyrinth disorders	Tinnitus, Hypoacusis	2
Endocrine disorders		
Eye disorders	Visual impairment, Vision blurred, Eye disorder	3
Gastrointestinal disorders	Abdominal distension, Abdominal pain, Gastrooesophageal reflux disease, Vomiting, Dyspepsia, Dysphagia, Gastrointestinal disorder, Nausea, Constipation, Abdominal discomfort, Diarrhoea, Abdominal pain upper, Flatulence, Dry mouth, Stomatitis	14
General disorders and administration site conditions	Peripheral swelling, Disease progression, Drug ineffective, Feeling abnormal, General physical health deterioration, Therapy non-responder, Drug intolerance, Asthenia, Death, Pyrexia, Malaise, Fatigue, Pain	13
Hepatobiliary disorders	Gallbladder disorder, Jaundice, Hyperbilirubinaemia	3
Immune system disorders	Graft *versus* host disease, Hypersensitivity	2
Infections and infestations	Nasopharyngitis, Upper respiratory tract infection, Pneumonia, Sinusitis, Sepsis, Urinary tract infection, Localised infection, Infection, Influenza, Bacteraemia, COVID-19	11
Injury, poisoning and procedural complications	Product dose omission issue, Fall, Contusion, Off label use	4
Investigations	Blast cell count increased, Platelet count decreased, Haemoglobin abnormal, White blood cell count increased, Full blood count abnormal, Haemoglobin decreased, Red blood cell count decreased, Weight decreased, Laboratory test abnormal, Weight increased, White blood cell count decreased, Platelet count abnormal, White blood cell count abnormal, Blood potassium decreased	14
Metabolism and nutrition disorders	Gout, Fluid retention, Dehydration, Decreased appetite, Tumour lysis syndrome	5
Musculoskeletal and connective tissue disorders	Pain in extremity, Musculoskeletal stiffness, Arthropathy, Muscle spasms, Myalgia, Arthralgia, Musculoskeletal pain, Bone pain, Back pain, Muscular weakness, Arthritis, Joint swelling, Back disorder	13
Neoplasms benign, malignant and unspecified (incl cysts and polyps)	Acute myeloid leukaemia recurrent, Differentiation syndrome, Acute myeloid leukaemia, Cholangiocarcinoma, Neoplasm malignant, Leukaemia recurrent, Malignant neoplasm progression	7
Nervous system disorders	Memory impairment, Hypoaesthesia, Paraesthesia, Neuropathy peripheral, Headache, Lethargy, Loss of consciousness, Seizure, Hypersomnia, Somnolence, Balance disorder, Dizziness	12
Pregnancy, puerperium and perinatal conditions		
Psychiatric disorders	Insomnia, Confusional state, Anxiety, Depression, Sleep disorder, Depressed mood	6
Renal and urinary disorders	Nephrolithiasis, Renal failure, Acute kidney injury, Chromaturia, Renal impairment, Renal disorder	6
Reproductive system and breast disorders		
Respiratory, thoracic and mediastinal disorders	Sinus disorder, Haemoptysis, Nasal congestion, Pulmonary oedema, Pleural effusion, Hypoxia, Epistaxis, Oropharyngeal pain, Respiratory failure, Cough, Dyspnoea	11
Skin and subcutaneous tissue disorders	Rash erythematous, Skin exfoliation, Pruritus, Dry skin, Urticaria, Rash, Erythema, Rash pruritic, Alopecia, Night sweats	10
Social circumstances	Loss of personal independence in daily activities	1
Surgical and medical procedures	Hospitalisation	1
Vascular disorders	Thrombosis, Hypotension, Haemorrhage	3
Blood and lymphatic system disorders	Cytopenia, Febrile neutropenia, Neutropenia, Anaemia, Haematotoxicity, Hyperleukocytosis, Lymphadenopathy, Leukocytosis, Pancytopenia, Thrombocytopenia, Blood disorder	11

When comparing the top 20 ADRs reported by two isocitratedehydrogenase inhibitor drugs, there are 22 differences at the PTs level (Table 7). Among them, the two drugs have the highest number of adverse reactions in injury, poisoning and procedural complexes, with a total of 22. The top five adverse reactions reported by enasidib are hip fracture, subdural haematoma, head injury, intentional product use issue and product use in unapproved indication. The top five adverse reactions reported by ivosidib are contracted product administered, product use issue, product administration error, product dose omission in error, product administration interrupted, followed by is nervous system disorders, with enasidib reporting the top five adverse reactions as cognitive disorder, taste disorder, dysgesia, ageusia, and amnesia; ivosidib reporting as the top five adverse reactions as syncope, migraine, guillain-barre syndrome, speech disorder and tremor.

**TABLE 7:** Different ADRs of two isocitrate dehydrogenase inhibitors drugs.

System organ classes	Enasidenib	Ivosidenib
Blood and lymphatic system disorders	Leukopenia, Disseminated intravascular coagulation, Thrombocytosis, Platelet disorder, Bone marrow failure, Bone marrow disorder, White blood cell disorder	Neutrophilia
Cardiac disorders	Cardiac arrest	Pericarditis, Acute myocardial infarction, Tachycardia, Bradycardia, Myopericarditis, Arrhythmia
Congenital, familial and genetic disorders		Janus kinase 2 mutation, Isocitrate dehydrogenase gene mutation
Ear and labyrinth disorders		Middle ear effusion, Vertigo
Endocrine disorders		
Eye disorders		Cataract, Dry eye, Diplopia, Photophobia
Gastrointestinal disorders	Frequent bowel movements, Oral pain, Gastrointestinal haemorrhage, Gastrointestinal pain	Faeces discoloured, Chapped lips, Bowel movement irregularity, Ascites, Retching
General disorders and administration site conditions	Multiple organ dysfunction syndrome, S welling, Illness, Adverse drug reaction, Oedema peripheral, Unevaluable event, Adverse event	Treatment noncompliance, Therapeutic response unexpected, Chills, Decreased activity, Chest pain, Drug interaction, Drug ineffective for unapproved indication
Hepatobiliary disorders	Liver disorder, Hepatotoxicity, Cholestasis, Hepatic cytolysis	Hepatic pain, Hepatic failure, Pseudocirrhosis
Immune system disorders	Immunodeficiency, Drug hypersensitivity, Seasonal allergy, Graft *versus* host disease in skin	
Infections and infestations	*Clostridium difficile* infection, Herpes zoster, Septic shock, Viral infection, Bronchitis, Staphylococcal infection, Coronavirus infection, COVID-19 pneumonia, Pneumonia fungal	Fungal infection, Fungaemia, Pneumonia parainfluenzae viral, Bronchopulmonary aspergillosis, Pharyngitis, Bacterial infection, Oral herpes, Cystitis, Diverticulitis
Injury, poisoning and procedural complications	Hip fracture, Subdural haematoma, Head injury, Intentional product use issue, Product use in unapproved indication, Transfusion reaction	Contraindicated product administered, Product use issue, Product administration error, Product dose omission in error, Product administration interrupted, Arthropod bite, Wrong technique in product usage process, Muscle strain, Toxicity to various agents, Extra dose administered, Prescribed underdose, Incorrect dose administered, Inappropriate schedule of product administration, Underdose, Intentional underdose, Product use complaint
Investigations	Neutrophil count decreased, Neutrophil count abnormal, Eastern cooperative oncology group performance status worsened, Blood bilirubin increased, Liver function test increased, Full blood count decreased	Electrocardiogram abnormal, Electrocardiogram qt prolonged, Heart rate increased, Blood test abnormal, Blood pressure increased, Red blood cell count abnormal
Metabolism and nutrition disorders		Hypokalaemia, Hyponatraemia, Malnutrition, Hyperkalaemia, Lactose intolerance, Hypophosphataemia, Increased appetite, Hypomagnesaemia
Musculoskeletal and connective tissue disorders	Myositis	Mobility decreased, Musculoskeletal discomfort, Muscle twitching, Muscle tightness, Flank pain
Neoplasms benign, malignant and unspecified (incl cysts and polyps)	Leukaemia, Acute myeloid leukaemia refractory, Myelodysplastic syndrome	Neoplasm, Glioblastoma multiforme, Neoplasm progression, Bone neoplasm, Marrow hyperplasia, Polycythaemia vera, Metastasis, Recurrent cancer
Nervous system disorders	Cognitive disorder, Taste disorder, Dysgeusia, Ageusia, Amnesia, Cerebral haemorrhage, Cerebrovascular accident, Dementia	Syncope, Migraine, Guillain-barre syndrome, Speech disorder, Tremor, Neuralgia, Burning sensation, Haemorrhage intracranial
Pregnancy, puerperium and perinatal conditions		
Product issues	Eating disorder	Emotional disorder, Listless, Stress, Hallucination, Emotional distress
Psychiatric disorders	Pollakiuria	Nocturia
Renal and urinary disorders		
Reproductive system and breast disorders	Lung disorder, Rhinorrhoea, Upper-airway cough syndrome, Pulmonary mass, Chronic obstructive pulmonary disease	Asthma, Acute respiratory failure, Choking, Dysphonia, Pulmonary embolism, Lung infiltration, Productive cough, Pneumonitis, Aphonia
Respiratory, thoracic and mediastinal disorders	Acute febrile neutrophilic dermatosis, Skin lesion, Skin disorder	Rash maculo-papular, Hyperhidrosis, Skin ulcer, Petechiae, Acne, Rash macular, Rash morbilliform
Skin and subcutaneous tissue disorders		
Social circumstances		Hospice care, Platelet transfusion, Hip arthroplasty, Therapy cessation, Bone marrow transplant, Transfusion
Surgical and medical procedures	Hypertension, Internal haemorrhage	Hot flush, Orthostatic hypotension
Vascular disorders	Leukopenia, Disseminated intravascular coagulation, Thrombocytosis, Platelet disorder, Bone marrow failure, Bone marrow disorder, White blood cell disorder	Neutrophilia

## 4 Discussion

Epigenetics mainly refers to heritable changes that regulate gene expression independent of DNA sequence changes and its mechanism mainly includes DNA methylation, histone modification, chromatin structure remodeling and non-coding RNA regulation (Gonzalez-Lugo et al., 2021).

Reports of mutations in genes encoding histone-modifying enzymes in cancers suggest that the global patterns of aberrant epigenetic modifications seen in some cancers may result from acquired mutations in genes that control this process. That these mutations are found in primary cancer cells implies that aberrant methylation contributes directly to tumor growth (Shannon and Armstrong, 2010).

In AML, some tumor suppressor genes are often hypermethylated, resulting in gene silencing, which can promote the proliferation and differentiation of leukemia cells. For example, mutations in DNMT3A are common in AML, which leads to changes in DNA methylation patterns and affects the gene expression. It often occurs that the histone methylation and other modifications abnormality in AML. These abnormal modifications affect chromatin structure and gene accessibility, and then affect gene expression. At the same time, epigenetic abnormalities can be used as an important indicator for the prognosis of AML. For example, IDH1/2 mutations may suggest a good prognosis in some cases. Targeted drugs aimed at epigenetic abnormalities provide a new idea for the treatment of AML.

Previous studies have found that the pathogenesis of acute myeloid leukemia (AML) is related to recurrent mutations that affect cellular metabolism and epigenetic regulation. About 10 years ago, recurrent somatic IDH1 and IDH2 gene mutations were discovered in AML with normal cytogenetics. The isoforms of IDH1 and IDH2 proteins play important parts in cellular metabolism and differentiation. α - KG is a recurrent hotspot mutation of the IDH1 and IDH2 genes, which is required for multiple critical dioxygenase reactions. It is now described in various cancers, including gliomas, chondrosarcomas and cholangiocarcinoma, and is most common in hematological malignancies, including myeloid malignancies (Abou Dalle and DiNardo, 2018).

Metabolism and epigenetics are highly interrelated. Mutations in genes encoding tricarboxylic acid cycle enzymes typically promote the development and progression of tumor by causing havoc with cellular metabolism and changing epigenetics. The subtype of isocitrate dehydrogenase (IDH1/2) is a typical example. The IDH enzyme metabolizes isocitric acid into α - ketoglutarate (α - KG). With the 2-HG increasing, α - KG levels decrease due to the functional IDH1 or IDH2mutations. α - KG works as an important cofactor for certain histones and DNA demethylases, while 2-HG is a competitive inhibitor that accumulates at high levels in cells, hindering the function of α - KG dependent enzymes (including epigenetic regulators), leading to histones and DNA hypermethylation, thereby altering gene expression and promoting cancer progression (Raineri and Mellor, 2018).

AML is one of the slowest progressing blood tumors in treatment research with no new drugs appearing for about 30 years. In 2017, there was a major outbreak of AML drug launches, and since then, the FDA has approved 7 AML drugs, including two IDH inhibitors, ivosidenib and enasidenib. Ivosidenib and enasidenib specifically inhibit the activity of mutated IDH1 and IDH2 enzymes respectively to lower the 2-HG levels, which helps to restore the differentiation process of cells and reduces the malignant proliferation of leukemia cells. Clinical trial results have shown that ivosidenib and enasidenib can significantly improve the survival rate and quality of life of AML patients with IDH1 and IDH2 gene mutations (Lee et al., 2019).

A clinical study showed that enasidenib can effectively inhibit the production of 2-HG in leukemia cell lines and induce cell differentiation in a dose-dependent manner. In the xenograft model, after treatment with enasidenib, the concentration of 2-HG in peripheral blood, bone marrow and spleen cells significantly decreased to near normal levels. In an invasive human AML xenograft mouse model, enasidenib was identified as having a dose-dependent survival advantage (Abou Dalle and DiNardo, 2018).

A study of persistent remission using ivosidenib in relapsed or refractory AML with IDH1 mutations showed that in (125 rate complete remission partial hematologic recovery was 30.4% (95% CI, 22.5–39.3), 21.6% CI, 14.7–29.8), overall response 41.6% 32.9–50.8) (DiNardo et al., 2018). These data indicated that ivosidenib had good tolerability and a high response rate in IDH1 mutant AML patients. Meanwhile, ivosidenib can be used to treat IDH1 mutant cholangiocarcinoma with significant survival benefits.

Although pre-market drug trials are very strict, due to the fact that these trials are conducted in controlled environments that are different from the actual usage environment, and clinical trials have certain intrinsic constraints, including strict experimental design, strict inclusion criteria, relatively small sample sizes and short follow-up times, it is impossible to fully understand the safety of drugs from preclinical trial data.

Spontaneous reporting systems (SRS) have been widely used for safety assessment of suspected adverse events in pharmacovigilance. Data from the SRS database can show the safety of specific drugs in real world better than clinical trials and plays an important role in signal recognition. At present, research on the safety signals of most drugs mainly comes from three main databases: the Eudra Vigilance Data Analysis System (EVDAS), Food and Drug Administration (FDA) Adverse Event Reporting System (FAERS), and WHO-VigiBase^®^ (Vogel et al., 2020). In 2015, WHO launched WHO vigiaccess to provide reports on potential side reactions of drugs to the public. Data mining of the WHO VigiAccess database will reveal some established clinical linkages and previously undiscovered drug AE associations. The study is aimed at evaluating the post marketing AEs associated with isocitrate dehydrogenase inhibitor drugs in the WHO VigiAccess database.

Due to strict data protection laws and agreements between WHO PIDM members and the WHO, individual case safety reports cannot be viewed in VigiAccess. VigiAccess groups the search results both by active ingredient and geographicaly by continental region so we are unable to retrieve data for specific brand names nor for individual WHO PIDM members. At the same time, we collected the data about two isocitrate dehydrogenase inhibitor drugs in WHO-Vigiaccess as much as possible, but some ADR reports lacked data on gender and age due to uncontrollable factors such as time and human factors. 81.4% of the adverse reaction reports of ivosidenib missed gender. Regarding age, 1,169 cases (42.1%) of enasidenib reports were lack of age, and 1,098 cases (84.7%) of ivosidenib reports were lack of age. When we analyze the adverse reactions caused by two isocitrate dehydrogenase inhibitor drugs on different gender or age groups, these missing data will inevitably affect the accuracy of our conclusions.

According to data from WHO VigiAccess, 87.3% of adverse event reports related to two types of isocitrate dehydrogenase inhibitor drugs came from America, followed by Europe at 11.3% and the lowest region was Asia at 0.1%. The estimated number of new diagnosed AML cases in America by the American Cancer Society is 20,240 in 2023, with the majority of patients being adults. Isocitrate dehydrogenase inhibitors have shown good efficacy in the treatment of AML patients with IDH mutations and have been widely used in the treatment of AML with IDH mutations resulting in the highest reported adverse events.

The reason why Asia had the lowest incidence of adverse events partly because most Asian regions are developing countries limited by medical standards, economic development, geography and social environment. For example, in lower-middle-income-countries such as India, the use of expensive drugs such as isocitrate dehydrogenase inhibitors is very low due to delayed diagnosis, increased infections, limited funding and limited access to new targeted treatment methods. Besides, Asian countries such as China usually have strict regulatory and approval processes for drugs, ensuring that marketed drugs meet certain standards in terms of safety, efficacy and quality. Taking China as an example, enasidenib was launched in the United States on 1 August 2017, but has not yet been officially launched in China. The launch date of ivosidenib in the United States 20 July 2018, while its launch date in China is 9 February 2022. It can be seen that the use of isocitrate dehydrogenase inhibitors in China is relatively short and has not yet been widely promoted and applied.

ADR report data shows that AE is more common in males than in females. The highest incidence of adverse events of isocitrate dehydrogenase inhibitors age group is ≥75 years, next is 65–74 years. This is mainly because these drugs have shown good efficacy and safety in clinical trials. Isocitrate dehydrogenase inhibitors are considered as first-line drugs especially in relapsed or refractory AML patients with IDH mutations. The incidence rate of AML is more than 65 years old and increases with age. As age increases, physiological functions gradually decline and the elderly have poor physical condition and drug tolerance, often accompanied by various underlying diseases. The low metabolic rate of drugs greatly increases the risk of adverse events. Therefore, although adverse events occur in all age groups, the incidence rate is highest in the age group ≥75 years old.

AE with a reporting rate of ≥1% is generally considered common (Chen et al., 2019). The serious AEs of two types of isocitrate dehydrogenase inhibitors, including death, hospitalization, life-threatening events, disability and congenital malformations. The death rate of enasidenib is 71.33%, much higher than that of ivosidenib. Under general disease and administration site conditions, these two isocitrate dehydrogenase inhibitors have the highest incidence of ADRs. The most frequent AEs of these two drugs are drug ineffective platelet count decreased, asthenia, fatigue and vomiting.

We performed enasidenib data mining in the FARES database to validate the results. The FAERS database is used for identifying potential association between drugs and adverse events in post-marketing surveillance of drug safety. However, there is a risk of bias due to the self-reported nature of the database. According to the requirements of regulatory agencies, the data in the FAERS database is anonymous. FDA publishes FAERS documents quarterly (i.e., 4 documents per year) (Peng et al., 2020). In our study, we extracted reports submitted between the first quarter of 2017 (FDA approved enasidenib) and the fourth quarter of 2023.

During the study period (the first quarter of 2017 and the fourth quarter of 2023), there were a total of 451 reports on enasidib. The clinical characteristics of enasidib events are shown in Table 8. Among all AEs, males (54.8%) accounted for a larger proportion than females who accepted manuscripts. The patients’ weight was mainly 50–100 kg (37.5%) and the main age was 65–85 (56.3%). AEs occurred mainly in the United States (22.0%). All individual AEs were determined based on MedDRA SOC and Pt levels recorded in the enasidenib report to describe the toxicity spectrum. When the number of cases is >3, the lower limit of the 95% confidence interval (CI) is >1.0, the ROR value is >2.0, and the Chi square value is >4.8, the ROR signal is positive (Sakaeda et al., 2013). An unexpected AE is defined as any significant AE found that is not listed in the FDA drug label. All data processing and statistical analysis were performed using R software (version 4.0.2).

**TABLE 8:** Characteristics of reports associated with enasidenib from August 2017 to December 2023.

	Enasidenib
Number of events	451
Gender	
Female	175 (38.8%)
Male	247 (54.8%)
Others	29 (6.4%)
Weight	
<50 kg	12 (2.7%)
>100 kg	10 (2.2%)
50∼100 kg	169 (37.5%)
Others	260 (57.6%)
Age	
<18	2 (0.4%)
18∼64.9	89 (19.7%)
65∼85	254 (56.3%)
>85	8 (1.8%)
Others	98 (21.7%)
Serious outcome	
Death	145 (32.2%)
Disability	4 (0.9%)
Hospitalization	184 (40.8%)
Life-threatening	44 (9.8%)
Reported countries	
Australia	41 (9.1%)
Germany	37 (8.2%)
France	87 (19.3%)
United States of America	99 (22.0%)
Others	187 (41.40%)

The significant SOCs were “Infections and infestations”, “Neoplasms benign, malignant and unspecified (incl cysts and polyps)” and “Blood and lymphatic system disorders” (Table 9), which was corresponding to previous safety data.

**TABLE 9:** Signal strength of AEs of enasidenib at the System Organ Class. Level in food and drug administration adverse event reporting system (FAERS) database.

System Organ Class	Enasidenib Cases Reporting SOC	ROR enasidenib/ all other cases(95%CI)
Nervous system disorders	34	0.56 (0.4–0.79)
Gastrointestinal disorders	62	0.97 (0.75–1.26)
General disorders and administration site conditions	86	0.57 (0.45–0.71)
Renal and urinary disorders	15	0.91 (0.55–1.52)
Infections and infestations	168	4.51 (3.81–5.35)
Metabolism and nutrition disorders	26	1.7 (1.15–2.51)
Neoplasms benign, malignant and unspecified (incl cysts and polyps)	109	4.08 (3.34–5)
Respiratory, thoracic and mediastinal disorders	55	1.56 (1.19–2.05)
Vascular disorders	7	0.46 (0.22–0.97)
Blood and lymphatic system disorders	87	7.1 (5.69–8.88)
Cardiac disorders	46	0.98 (0.73–1.32)
Injury, poisoning and procedural complications	19	1.2 (0.76–1.89)
Investigations	22	0.21 (0.14–0.32)
Musculoskeletal and connective tissue disorders	11	1.68 (0.93–3.05)
Skin and subcutaneous tissue disorders	16	0.33 (0.2–0.54)
Immune system disorders	14	0.33 (0.2–0.56)

CI, confidence interval; ROR, reporting odds ratio.

By comparing the adverse reaction reporting data of enasidenib in the WHO- Vigiaccess and FARES databases, we found that there were gender differences in the adverse reactions of enasidenib. The rate of adverse reactions in men was higher than that in women, and they were more common in the elderly. The adverse reaction reporting rate in the United States was significantly higher than that in other countries. The serious consequences of enasidenib leading to patient death were reported at a high rate. However, different from the data mining results of WHO- Vigiaccess, the SOC signal of infections and infestations caused by enasidenib in FARES database was stronger, which needed to cause pharmacovigilance.

A recent FDA systematic analysis reported that the incidence rate of DS was 19%. A clinical study showed that the most frequent AEs associated with using enasidenib were indirect hyperbilirubinemia (40.3%), nausea (28%) and decreased appetite (17.7%). The most common grade 3 or 4 treatment adverse events related to enasidenib are hyperbilirubinemia (10.4%), thrombocytopenia (6.7%), IDH differentiation syndrome (IDH-DS; 6.4%) and anemia (5.5%) (Stein et al., 2019). IDH-DS patients may have mild to moderate symptoms, including unexplained fever, edema or creatinine changes. However, critically ill patients may experience severe respiratory and hemodynamic damage, requiring hospitalization and admission to the intensive care unit, with the most common being respiratory distress and lung infiltration (Montesinos et al., 2024). The possible reason is that isocitrate dehydrogenase inhibitor drugs induce terminal differentiation of AML cells by targeting IDH2 receptors, thereby inducing the production of chemokines in the lungs, leading to the migration, adhesion and infiltration of differentiated cells into the lungs and other tissues. These chemokines can also act as chemotactic agents for other inflammatory cells, further aggravating the high inflammatory state. Therefore, when using isocitrate dehydrogenase inhibitor drugs in clinical practice, clinical doctors should closely monitor the early symptoms and signs of DS reported, which may also occur after treatment interruption and restarting medication. Early identification of DS using standardized diagnosis is helpful for early diagnosis and treatment. IDH DS can be treated with dose interruption and corticosteroids, oral hydroxyurea or both simultaneously (Carter et al., 2020).

The most common ADRs of the two drugs in our study were blood and lymphatic system disorders. Ivosidenib and enasidenib are both targeted drugs that exert therapeutic effects by inhibiting specific enzymes or signaling pathways. These enzymes or signaling pathways play critical roles in processes such as cell proliferation, differentiation, and apoptosis, particularly in the generation and function of blood cells and lymphocytes (Shannon and Armstrong, 2010). Therefore, when these drugs inhibit these critical pathways, they may affect the normal physiological functions of blood and lymphocytes, leading to blood and lymphatic system diseases. During the metabolism and excretion of drugs in the body, harmful metabolites or toxic substances may be produced, which can directly damage biomolecules such as DNA, RNA or proteins in blood cells or lymphocytes, leading to abnormal cell function or death.

We analyzed the different points in ADRs between these drugs. The two drugs have the highest incidence of adverse reactions in injury, poisoning and procedural complications. The main adverse reactions reported by enasidib are hip fracture, subdural haematoma and head injury, while the main adverse reactions reported by ivosidib are contracted product administered, product use issue and product administration error. These differences of ADRs may be associated with the different molecular weight, structure, mechanism or pharmacokinetics of the drug.

The evaluation of whether the combination therapy of ivosidenib and enasidenib can be applied in clinical practice is still ongoing. The latest results shows that the combination of ivosidenib and enasidenib with standard DA regimen has achieved good efficacy in the treatment of newly diagnosed AML patients with IDH1 or IDH2 mutations. In another study on the combination of ivosidenib or enasidenib with intensified chemotherapy for newly diagnosed AML patients, the induced final CR and CR/CRI/CRp rates in the ivosidenib group were 55% and 72% respectively, while the induced final CR and CR/CRI/CRp rates in the enasidenib group were 47% and 63% respectively. The optimal total CR and CR/CRI/CRp rates for patients treated with ivosidenib were 68% and 77%, respectively, while those for patients treated with enasidenib were 55% and 74%, respectively (Stein et al., 2021). A Phase 3 study further confirmed the efficacy and safety of ivosidenib combined with azacitidine in patients with AML who are not suitable for intensified chemotherapy. Compared with placebo combined with azacitidine, ivosidenib plus azacitidine significantly prolonged event free survival and overall survival, and responded well (Cai et al., 2024). Although ivosidenib and enasidenib are currently approved as monotherapy, the use of well tolerated and reasonable combination therapy will undoubtedly further improve persistent patient response and increasingly improve patient prognosis.

ADR report data showed that adverse drug reactions were more frequent in men than in women and the age group with the highest incidence of adverse events was ≥75 years old. Therefore, for elderly patients with AML, especially male patients, the dose of isocitrate dehydrogenase inhibitor can be appropriately reduced to decrease the occurrence of adverse reactions while maintaining the efficacy.

The most common adverse reactions of these two drugs are drug ineffective and platelet count decreased. For patients with drug ineffective and their symptoms have not improved after using drugs for a period of time, they should use another drug in time. If the drug causes platelet count decreased, the blood routine should be reviewed regularly and patients should stop using the drug or use another drug when necessary. When the platelet count is severely reduced and accompanied by bleeding, it is recommended to carry out platelet transfusion under the guidance of doctors. The mortality rate of enasidenib was 71.33%, which was much higher than that of ivosidenib, indicating that when using enasidenib clinically, doctors should be more cautious in prescribing the dose and pay more attention to the performance of patients after using it.

IDH inhibitors provide a new treatment option for specific types of cancer patients, especially AML patients with IDH mutations. They have shown good efficacy in tumor treatment, which can effectively prolong patients’ survival and improve their quality of life, but they may also lead to some adverse reactions. By effectively managing ADR, on the one hand, we can reduce the occurrence of the adverse reactions to enhance the tolerance and compliance of patients. On the other hand, this also helps to optimize treatment plans, ensuring that patients achieve optimal treatment outcomes while minimizing the occurrence of adverse reactions, thereby improving the therapeutic effect of IDH inhibitors.

Of course, SRS has certain limitations, as reports may be affected by uncertain factors such as reputation bias, selection bias, and underreporting. From the current reports of AE research results, it is observed that the missing data cannot be attributed to either males, females, or age groups. Meanwhile, since the VigiAccess database of the WHO is cumulative data, the ADRs of every year cannot be obtained. When drugs are put on the market at different times, the number of ADRs collected is quite different, and the signal difference of all target inhibitors cannot be compared at the same time (Li et al., 2023). Moreover, we are unable to acquire all AEs related to specific drugs through VigiAccess.

This study gathered the quantity of ADRs and PTs from 2017 to 2024 and avoided the influence of drug approved marketing time by comparing the ADR reporting rates of two drugs. The research results are limited to the relative results of two isocitrate dehydrogenase inhibitors and we need further clinical studies to provide stronger evidence in our real world.

## 5 Conclusion

Isocitrate dehydrogenase inhibitors are widely used for treating AML with IDH mutations. Research shows that WHO-VigiAccess reported 4,072 cases of adverse reactions caused by treatment with isocitrate dehydrogenase inhibitors. The adverse reactions of these drugs are mainly concentrated in general disorders and administrative site conditions, injury poisoning and procedural complications, investigations and gastrointestinal disorders. It reconfirms the adverse reaction symptoms of injury poisoning, procedural complications and gastrointestinal disorders.

In addition, the neoplastics benign and non-specific inward cysts and polyps caused by enasidib, as well as the nervous system disorders caused by ivosidib, are also very prominent. Although most adverse reactions of drugs are slight and self-limited, there are also some serious adverse reactions that may result in hospitalization, life-threatening situations or even death for patients. Therefore, in clinical applications, clinical doctors should pay attention to common ADRs and be alert to the occurrence of serious ADRs. If necessary, patients should stop taking medication in a timely manner to avoid fatal ADRs.

It is concluded from our study that the adverse reactions of drugs should be analyzed on the basis of extensive promotion and application of drugs, taking the medical level, economic development, geographical and social environment constraints of different countries and regions into consideration, so as to make the research results representative and meaningful. At the same time, countries around the world should actively carry out the safety research on biologics to study the causal relationship between ADRs and medications. The research results can be stored in open access databases to strengthen public understanding of the side effects of biotechnology drugs. Future drug research strategies should focus on the development of rational combination therapy with IDH1/2 inhibitors and other effective therapies to provide more possibilities for the treatment of AML.

## Author contributions

MP: Writing–original draft, Visualization, Validation, Formal Analysis, Data curation, Conceptualization. QG: Formal Analysis, Data curation, Writing–original draft. ZD: Formal Analysis, Data curation, Writing–original draft. BZ: Formal Analysis, Data curation, Writing–original draft. ML: Visualization, Validation, Writing–review and editing, Writing–original draft. ZW: Visualization, Validation, Writing–original draft. XL: Supervision, Project administration, Methodology, Writing–review and editing, Writing–original draft. JL: Supervision, Project administration, Methodology, Funding acquisition, Conceptualization, Writing–review and editing, Writing–original draft.

## Data Availability

The original contributions presented in the study are included in the article/supplementary material, further inquiries can be directed to the corresponding authors.
